# Cytosolic Phospholipase A_2_ alpha/Arachidonic Acid Signaling Mediates Depolarization-Induced Suppression of Excitation in the Cerebellum

**DOI:** 10.1371/journal.pone.0041499

**Published:** 2012-08-22

**Authors:** De-Juan Wang, Dong Yang, Li-Da Su, Ya-jun Xie, Lin Zhou, Cheng-Long Sun, Yin Wang, Xin-Xin Wang, Liang Zhou, Ying Shen

**Affiliations:** 1 Key Laboratory of Medical Neurobiology of the Ministry of Health, Zhejiang Province Key Laboratory of Neurobiology, Department of Neurobiology, Zhejiang University School of Medicine, Hangzhou, People's Republic of China; 2 Neuroscience Care Unit, The Second Affiliated Hospital of Zhejiang University School of Medicine, Hangzhou, People's Republic of China; 3 Department of Neurobiology, Center of Scientific Technology, Cranial Cerebral Disease Lab, Ningxia Medical University, Yinchuan, People's Republic of China; National Institute of Health, United States of America

## Abstract

**Background:**

Depolarization-induced suppression of excitation (DSE) at parallel fiber-Purkinje cell synapse is an endocannabinoid-mediated short-term retrograde plasticity. Intracellular Ca^2+^ elevation is critical for the endocannabinoid production and DSE. Nevertheless, how elevated Ca^2+^ leads to DSE is unclear.

**Methodology/Principal Findings:**

We utilized cytosolic phospholipase A_2_ alpha (cPLA_2_α) knock-out mice and whole-cell patch clamp in cerebellar slices to observed the action of cPLA_2_α/arachidonic acid signaling on DSE at parallel fiber-Purkinje cell synapse. Our data showed that DSE was significantly inhibited in cPLA_2_α knock-out mice, which was rescued by arachidonic acid. The degradation enzyme of 2-arachidonoylglycerol (2-AG), monoacylglycerol lipase (MAGL), blocked DSE, while another catabolism enzyme for N-arachidonoylethanolamine (AEA), fatty acid amide hydrolase (FAAH), did not affect DSE. These results suggested that 2-AG is responsible for DSE in Purkinje cells. Co-application of paxilline reversed the blockade of DSE by internal K^+^, indicating that large conductance Ca^2+^-activated potassium channel (BK) is sufficient to inhibit cPLA_2_α/arachidonic acid-mediated DSE. In addition, we showed that the release of 2-AG was independent of soluble NSF attachment protein receptor (SNARE), protein kinase C and protein kinase A.

**Conclusions/Significance:**

Our data first showed that cPLA_2_α/arachidonic acid/2-AG signaling pathway mediates DSE at parallel fiber-Purkinje cell synapse.

## Introduction

Depolarization-induced suppression of excitation (DSE) was first reported at excitatory synapse in cerebellar Purkinje cells [1]. While DSE is a short-term retrograde plasticity associated with a change in paired-pulse ratio [1]–[3], it is initiated by the postsynaptic depolarization that activates local dendritic Ca^2+^ spikes [1], [4]. Both blocking dendritic Ca^2+^ spikes by hyperpolarization and intracellular injection of 1,2-bis(o-aminophenoxy)ethane-N,N,N′,N′-tetraacetic acid (BAPTA) prevent DSE [1], [4], indicating that the Ca^2+^ elevation is critical for the DSE induction. DSE provides a means for altering the strength and properties of presynaptic inputs for tens of seconds during high postsynaptic activity [1]. It is postulated that DSE provides a neuroprotective effect because it reduces the glutamatergic transmission when Purkinje cells are subject to strong excitatory inputs in pathophysiological conditions [1]–[3].

It is known that DSE is mediated by a retrograde signaling that involves the production of postsynaptic endocannabinoid and the activation of presynaptic cannabinoid receptor 1 (CB1R) [2], [3]. The synthesis and release of endocannabinoids are Ca^2+^-dependent [5], [6]. Nevertheless, how Ca^2+^ elevation leads to the production of endocannabinoid is unclear thus far. It is shown that a prolonged elevation of synaptic Ca^2+^ activates Gq-coupled metabotropic receptors [7], [8] and phospholipase-C (PLC) [6]. However, this may not be the case for DSE induction in Purkinje cells, because DSE is independent of PLC [9] and metabotropic glutamate receptor (mGluR) [1]. Thus, another PLC-independent pathway might contribute to the production of endocannabinoid and DSE induction in Purkinje cells.

The phospholipase A_2_ (PLA_2_) enzymes catalyze ester hydrolysis of fatty acids [10]. Of the PLA_2_s, cytosolic phospholipase A_2_ alpha (cPLA_2_α) has a unique set of biochemical properties. It translocates to cellular membranes in response to micromolar intracellular Ca^2+^ and produces arachidonic acid [11]. Arachidonic acid can be metabolized by a number of enzymes to create the eicosanoids [10], [12] that play important roles in regulating cellular homeostasis, neurotoxicity and inflammation [11]–[13]. Since the brief depolarization during DSE exerts a rapid elevation of intracellular Ca^2+^ with a peak level of 10–15 µM [8], [14], we hypothesized that the elevated Ca^2+^ triggers the activation of cPLA_2_α and causes the synthesis and release of endocannabinoid. Here, we examined the function of cPLA_2_α/arachidonic acid pathway in DSE at parallel fiber-Purkinje cell synapses derived from wild-type (WT) and cPLA_2_α knock-out (KO) mice. We also explored other unsolved mechanisms of DSE in Purkinje cells using various pharmacological treatments. In summary, our data showed that the cPLA_2_α/arachidonic acid pathway is required for DSE induction.

## Results

### DSE is inhibited in cPLA_2_α KO mice

**Figure 1:**
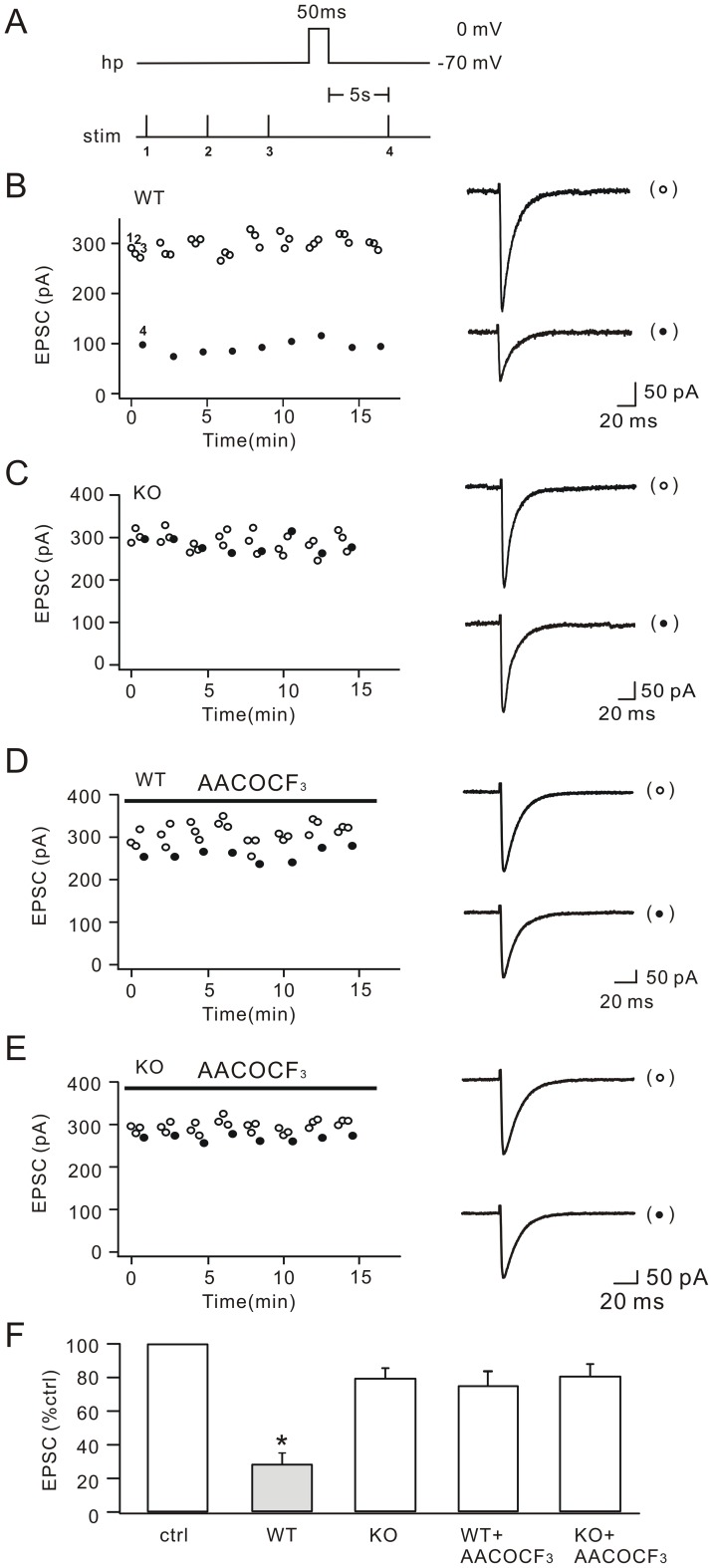
cPLA_2_α deficiency inhibits DSE at parallel fiber-Purkinje cell synapse. (A) The stimulus protocol with the holding potential (hp) of Purkinje cells and the stimulation timing (stim). The duration of depolarization to 0 mV was 50 ms. Δt between the depolarization and the test stimulus was 5 s. The numbers 1, 2, 3 index control parallel fiber stimuli and 4 labels the test stimulation. The intervals between indexed 1, 2, 3 were 20 s. The intervals between 3 and depolarization was 10 s. (B) Amplitudes of parallel fiber EPSCs derived from one WT Purkinje cell plotted over time for control responses with no preceding prepulse to 0 mV (open circles) and test responses following depolarization (closed circles). Numbered circles (1, 2, 3, 4) correspond to the control and test stimuli in (A), respectively. Representative EPSCs are shown at the right. Stimulus artifacts are blanked for clarity. (C) EPSCs of one KO Purkinje cell plotted over time for control responses with no preceding prepulse to 0 mV (open circles) and test responses following depolarization (closed circles). Representative EPSCs are shown at the right. (D) EPSCs derived from one WT Purkinje cell plotted over time. AACOCF_3_ was perfused throughout the experiment, as shown by the bar at top. Control and test responses are shown by open and closed circles, respectively. Representative EPSCs are shown at the right. (E) EPSCs derived from one KO Purkinje cell plotted over time. AACOCF_3_ was perfused throughout the experiment, as shown by the bar at top. Control and test responses are shown by open and closed circles, respectively. Representative EPSCs are shown at the right. (F) Bar graphs show the percentage inhibitions of test EPSCs in WT, KO, WT+AACOCF_3_ and KO+AACOCF_3_. ctrl: control responses (n = 88). WT: 28.3±5.4%; n = 26. KO: 79.4±5.8%; n = 20. WT+AACOCF_3_: 75.7±8.3%; n = 22. KO+AACOCF_3_: 80.7±6.7%; n = 20. *, P<0.05.

DSE at the parallel fiber-Purkinje cell synapse was studied in sagittal cerebellar slices. Parallel fiber excitatory postsynaptic currents (EPSCs) were evoked with an extracellular electrode placed in the molecular layer. DSE was induced according to the previous work [1]. In brief, Purkinje cells were stimulated by a step voltage from −70 mV to 0 mV (50 ms) after 3 consecutive control EPSCs with an interval of 20 s were obtained in voltage-clamp mode (Figure 1A). A test stimulus was set to 5 s after the depolarization to acquire the test EPSC. In WT mice, the amplitudes of test EPSCs were greatly reduced and the EPSC inhibition usually recovered within 90 s (Figure 1B). We could reliably repeat this cycle every 90 s (Figure 1B), similar to previous work [1]. To confirm the validity of our DSE experiment, we also perfused AM251, a CB1R antagonist, in cerebellar slices. We found that the inhibition of test EPSC was completely blocked by AM251 (Figure S1), demonstrating that our DSE experiment was successful [1], [4], [15]. Interestingly, we found that DSE was significantly reduced in KO mice (Figure 1C). Pretreatment of WT cells with the cPLA_2_α inhibitor arachidonyl trifluoromethyl ketone (AACOCF3; 10 µM) also resulted in a significant reduction of DSE (Figure 1D). Finally, AACOCF3′s effect was examined in KO cells. Similar to WT cells, we failed to observe DSE (Figure 1E). The statistics are shown in Figure 1F, indicating that cPLA_2_α is required for DSE.

### Arachidonic acid rescues DSE in cPLA_2_α KO mice

**Figure 2:**
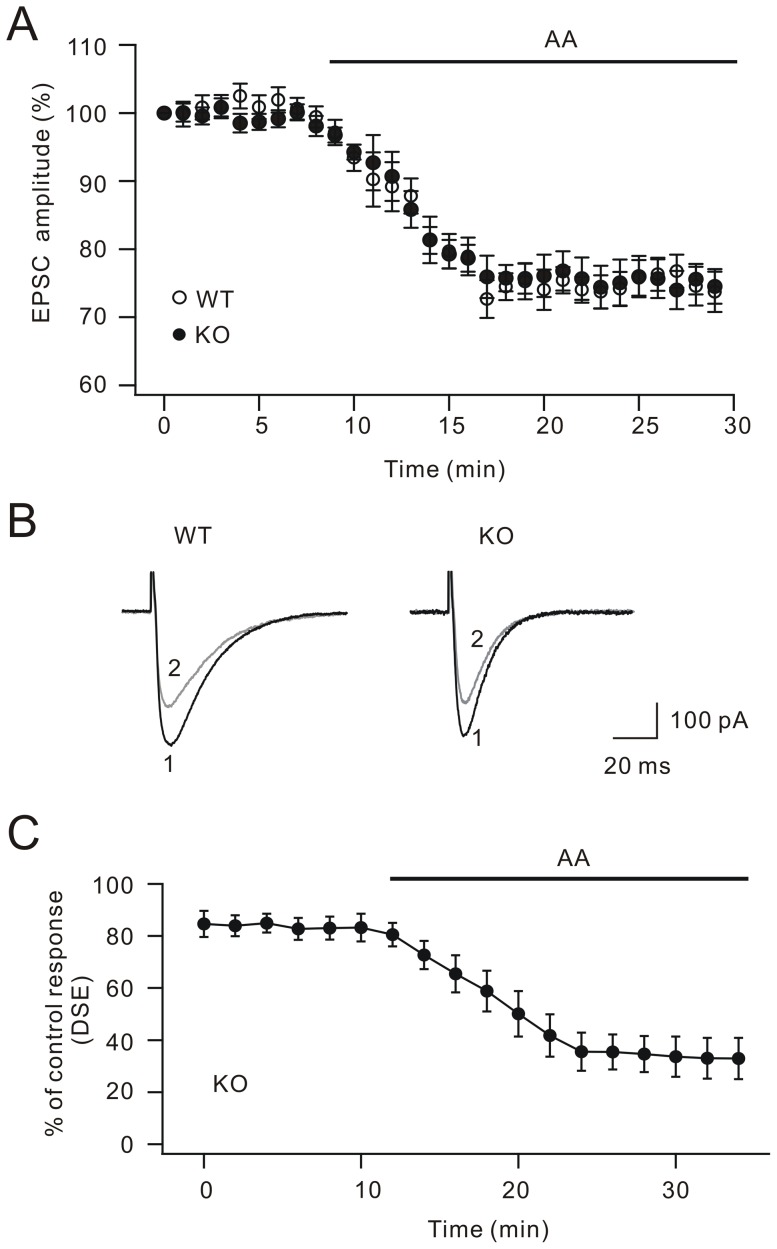
Arachidonic acid rescues DSE in cPLA_2_α knock-out mice. (A) Time courses of percentage changes of parallel fiber EPSC amplitudes derived from WT (open circles) or KO (filled circles) mice. Arachidonic acid was applied in the bath as indicated by the bar. Arachidonic acid depressed EPSCs in both KO and WT cells. (B) Representative parallel fiber EPSCs from WT and KO cells at the time points indicated in (A). Stimulus artifacts are blanked for clarity. (C) Arachidonic acid restored DSE in KO cells. DSE was induced by the protocol indicated in Figure 1A with Δt 5 s. Each data point represents the percentage inhibition of test EPSC every 2 min. Arachidonic acid was applied in the bath as indicated by the bar.

cPLA_2_α selectively liberates arachidonic acid [11]. We next examined the effects of external application of arachidonic acid in parallel fiber EPSC and DSE. Parallel fiber-Purkinje cell synaptic EPSCs were evoked and recorded every 20 s. Bath application of arachidonic acid (10 µM) decreased EPSCs in WT Purkinje neurons (73.7±3.0% of baseline at t = 29 min; n = 25; Figure 2A). Similarly, 10 µM arachidonic acid induced a slow decrease of EPSCs that reached 74.5±2.5% of baseline at t = 29 min in KO cells (n = 17; Figure 2A). Representative recordings are shown in Figure 2B. These data implied that arachidonic acid, as the downstream factor of cPLA_2_α, is able to suppress parallel fiber EPSC. We next asked whether exogenous arachidonic acid is able to rescue the defect of DSE in KO Purkinje cells. In this experiment, DSE was evoked every 2 min using the protocol in Figure 1A. The ratio of test/control response was gradually reduced in the bath application of arachidonic acid (33.0±7.9% at t = 34 min; n = 19; Figure 2C), indicating that arachidonic acid is able to rescue DSE at parallel fiber-Purkinje cell synapse. Collectively, these experiments demonstrated that cPLA_2_α/arachidonic acid signaling pathway is essential to the induction of DSE in Purkinje cells.

### 2-AG is the downstream factor of arachidonic acid in DSE

**Figure 3:**
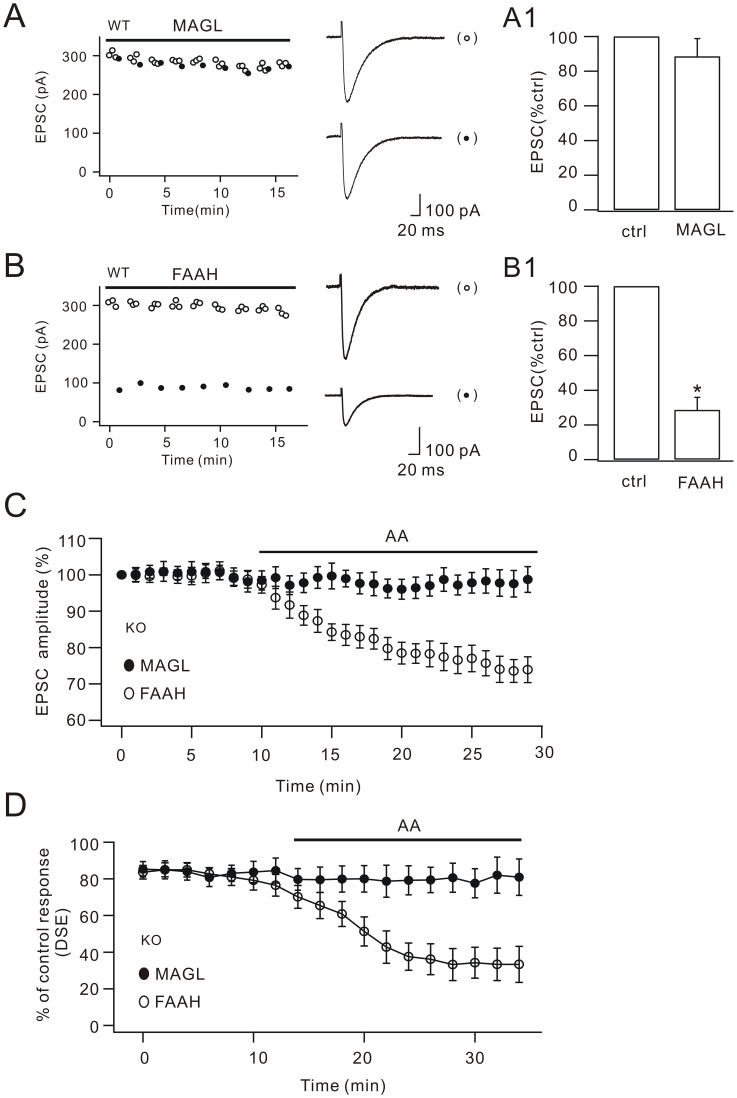
MAGL blocks the action of arachidonic acid in DSE. (A) EPSCs from one WT Purkinje cell plotted over time for control responses (open circles) and test responses (closed circles). Cells were filled with MAGL as indicated by the bar. Representative EPSCs are shown at the right. The percentage inhibition of test EPSCs (89.7±9.1%; n = 21) is shown in (A1). (B) EPSCs from one WT Purkinje cell plotted over time for control (open circles) and test responses (closed circles). Cells were filled with FAAH as indicated by the bar. Representative EPSCs are shown at the right. The percentage inhibition of test EPSCs (28.7±7.1%; n = 23) is shown in (B1). (C) Time courses of percentage changes of parallel fiber EPSC amplitudes derived from KO cells filled with either MAGL (filled circles) or FAAH (open circles). Arachidonic acid (AA) was applied in the bath as indicated by the bar. Arachidonic acid depressed EPSCs in FAAH-filled cells but not MAGL-filled cells. (D) MAGL blocked the rescue of DSE by arachidonic acid in KO cells. KO cells filled with either MAGL (filled circles) or FAAH (open circles) Arachidonic acid restored DSE in KO cells. DSE was induced by the protocol indicated in Figure 1A with Δt 5 s. Each data point represents the average percentage inhibition of test EPSC every 2 min. Arachidonic acid was applied in the bath as indicated by the bar. *, P<0.05.

N-arachidonoylethanolamine (AEA) and 2-arachidonoylglycerol (2-AG) are two endogenous ligands that activate CB1Rs [16]. The biosynthetic processes and catabolic inactivation of AEA and 2-AG decide the onset and duration of CB1R-mediated signaling [6], [16]. In central nervous system (CNS), AEA is selectively hydrolyzed by fatty acid amide hydrolase (FAAH) [17], [18] and 2-AG is selectively hydrolyzed by monoacylglycerol lipase (MAGL) [19], [20]. We added 0.7 µg/ml MAGL or 1 µg/ml FAAH in the pipette solution during the DSE experiments in WT cells, according to previous work [21]. These operations broke down 2-AG and AEA respectively in Purkinje cells [21]. As shown in Figure 3A and B, DSE was blocked in the presence of MAGL, but kept unchanged in the presence of FAAH, indicating that 2-AG but not AEA is required for DSE.

We showed that arachidonic acid is able to decrease parallel fiber EPSC and rescue DSE (Figure 2). Next, we asked whether arachidonic acid's roles are mediated by 2-AG. A baseline of parallel fiber EPSCs was recorded in KO cells that were filled with 0.7 µg/ml MAGL and 1 µg/ml FAAH (Figure 3C). We then applied exogenous arachidonic acid in order to examine whether it affects EPSC when 2-AG or AEA is broken down. EPSCs obtained from FAAH-filled KO cells decreased after 10 µM arachidonic acid was applied (73.9±3.7% of baseline at t = 29 min; n = 17; Figure 3C). On the contrary, arachidonic acid did not change parallel fiber EPSCs in MAGL-filled KO cells (98.7±3.6% of baseline at t = 29 min; n = 19; Figure 3C). These results suggested that the arachidonic acid's suppression of EPSC depends on the production of 2-AG. We continued to study whether MAGL or FAAH affects the rescue of DSE by arachidonic acid in KO cells. In this experiment, DSE was assessed every 2 min from either 0.7 µg/ml MAGL-filled or 1 µg/ml FAAH-filled KO cells. After a baseline recording of DSE, 10 µM arachidonic acid was perfused in slices. We found that exogenous arachidonic acid failed to produce DSE in MAGL-filled KO cells (ratio of test/control response: 81.0±9.9% at t = 34 min; n = 20; Figure 3D). However, arachidonic acid effectively rescued DSE in FAAH-filled KO cells (ratio of test/control response: 33.3±9.8% at t = 34 min; n = 18; Figure 3D). These results indicated that roles of cPLA_2_α/arachidonic acid in DSE are *via* 2-AG but not AEA. Interestingly, we found that DSE was gradually increased with the application of arachidonic acid in KO cells (Figure 2C and Figure 3D). This implied that the depolarization not only activates cPLA_2_α, but facilitates the release of 2-AG. Indeed, the suppression ratios of test EPSC *vs*. control EPSC in DSE (Figure 2C and Figure 3D) were much more prominent than the decreases of EPSC caused by arachidonic acid application (Figure 2A and 3C).

### Ca^2+^-activated K^+^-channel inhibitor paxilline reverses the blockade of DSE by internal K^+^

**Figure 4:**
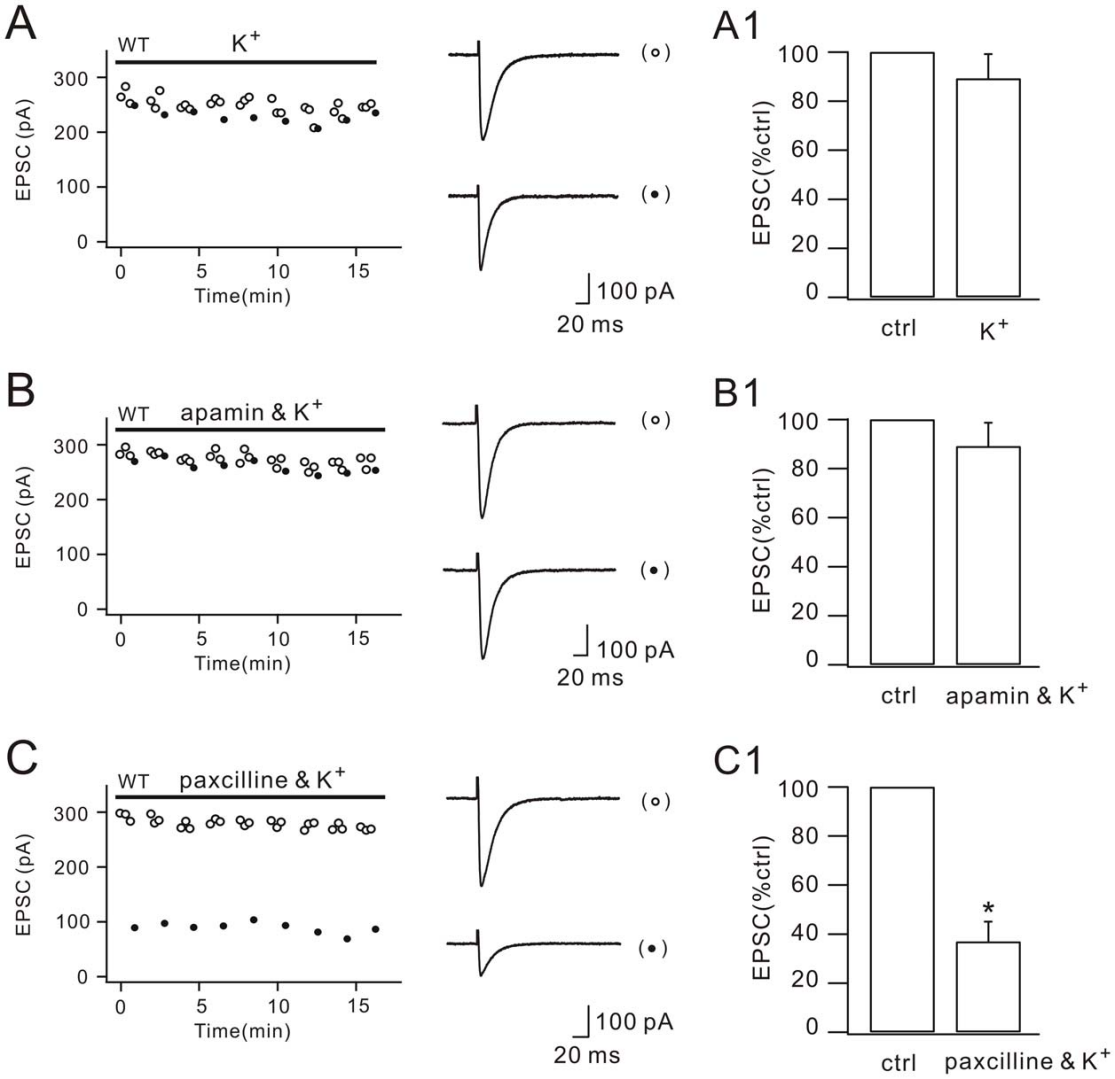
Paxilline reverses the blockade of DSE by internal K^+^. (A) EPSCs from one WT Purkinje cell plotted over time for control responses (open circles) and test responses (closed circles). Representative EPSCs are shown at the right. Internal K^+^ was applied as indicated by the bar. The percentage inhibition of test EPSCs (89.3±10.4%; n = 26) is shown in (A1). (B) EPSCs from one WT Purkinje cell plotted over time for control (open circles) and test responses (closed circles). Representative EPSCs are shown at the right. Internal K^+^ plus external apamin was applied as indicated by the bar. The percentage inhibition of test EPSCs (89.2±9.9%; n = 20) is shown in (B1). (C) EPSCs from one WT Purkinje cell plotted over time for control (open circles) and test responses (closed circles). Representative EPSCs are shown at the right. Internal K^+^ plus external paxilline was applied as indicated by the bar. The percentage inhibition of test EPSCs (36.6±8.4%; n = 22) is shown in (C1). *, P<0.05.

Our findings suggested that the depolarization-induced calcium increase is sufficient to activate the cPLA_2_α/arachidonic acid pathway, consistent with the characteristic low-threshold activation of cPLA_2_α [10], [11]. Previous work showed that voltage-gated calcium channels (VGCCs) attribute the most of calcium influx [4], but how Ca^2+^ influx is regulated in DSE is unclear. Although hyperpolarization in Purkinje cells prevents the induction of DSE [4], [14], the function of K^+^ channels in DSE is unknown because Cs^+^-based internal saline was used in previous studies [1], [4], [14], [15]. To examine the role of K^+^ channels in cPLA_2_α activation and DSE, we switched the internal saline from Cs^+^-based to K^+^-based and examined DSE in WT cells. We found that DSE was completely blocked (Figure 4A), suggesting that the activation of K^+^ channels are sufficient to inhibit Ca^2+^ influx and DSE.

We next studied which K^+^ channel was involved in the inhibition of Ca^2+^ influx. A number of conductances contribute to spike repolarization in Purkinje neurons, including large-conductance calcium-activated potassium channel (BK) and small-conductance calcium-activated potassium channel (SK). If the opening of BK or SK weakens Ca^2+^ influx and inhibits DSE, then a pharmacologic disruption of BK and SK should reverse the blockade of DSE. Accordingly, we used Cs^+^-based internal saline in pipettes and perfused WT cells with either BK blocker, paxilline (1 µM), or SK blocker, apamin (100 nM). As shown in Figure 4B, co-application of apamin did not induce DSE. On the contrary, co-application of paxilline relieved the blockade of DSE by internal K^+^ (Figure 4C), indicating that BK opening is sufficient to block the internal Ca^2+^-induced cPLA_2_α inactivation and DSE induction.

### Beyond cPLA_2_α/arachidonic acid: DSE is independent of SNARE, P2X7 receptor (P2X7R), protein kinase C (PKC) and protein kinase A (PKA)

**Figure 5:**
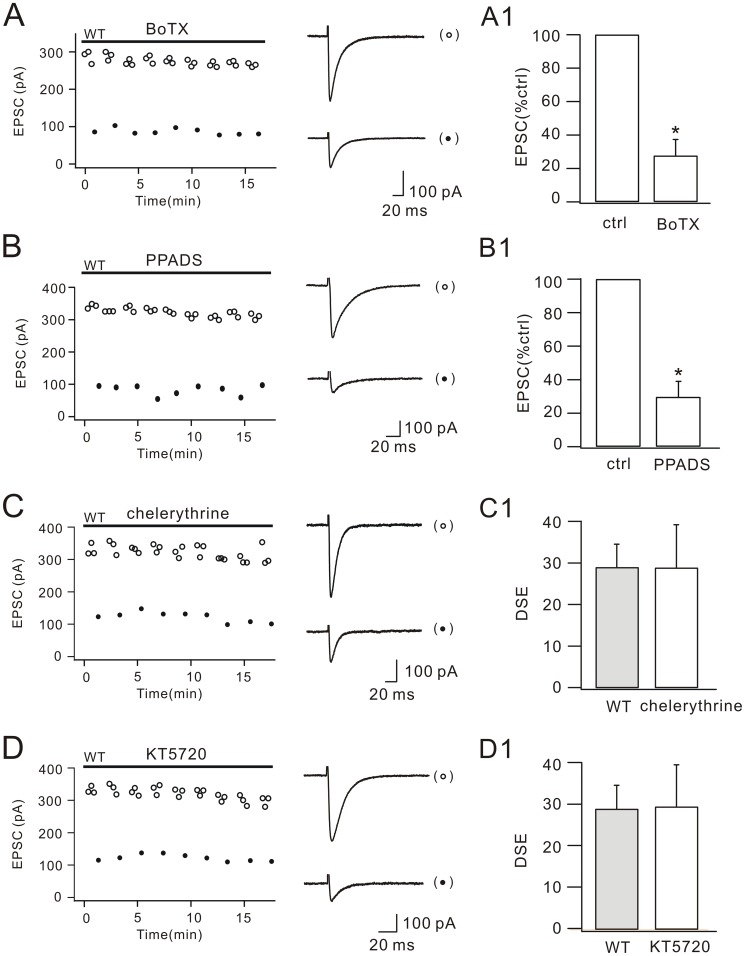
BoTx, PPADS, chelerythrine and KT5720 do not influence DSE. (A) EPSCs from one WT Purkinje cell plotted over time for control (open circles) and test responses (closed circles). Representative EPSCs are shown at the right. Internal BoTx was applied as indicated by the bar. The percentage inhibition of test EPSCs (27.8±9.5%; n = 17) is shown in (A1). (B) EPSCs from one WT Purkinje cells plotted over time for control (open circles) and test responses (closed circles). Representative EPSCs are shown at right. The percentage inhibition of test EPSCs (29.2±9.1%; n = 25) is shown in (B1). (C) and (D), Control (open circles) and test (closed circles) EPSCs from two WT Purkinje cells are plotted over time. Representative EPSCs are shown at the right. Representative EPSCs are shown at the right. (C1) and (D1) show DSE amplitudes in chelerythrine (28.7±10.3%; n = 18) and KT5720 (29.2±10.1%; n = 18), respectively. DSE in WT cells (Figure 1E; gray bar) is replotted in (C1) and (D1) for comparison. Applications of BoTx, PPADS, chelerythrine and KT5720 are indicated by bars. Stimulus artifacts of EPSCs are blanked for clarity. *, P<0.05.

Although we showed that cPLA_2_α/arachidonic acid signaling was essential for DSE induction, several important questions in Purkinje cell DSE remain to be elucidated. Previous work showed that the production site of endocannabinoid and presynaptic CB1 receptors are in close proximity of postsynaptic neurons [22]. Thus, the first question is how endocannabinoid (2-AG) is released. Most retrograde messengers are stored in vesicles and released through exocytosis. Is 2-AG released *via* secretory vesicles at parallel fiber-Purkinje cell synapse? Botulinumtoxin (BoTx) destroys the stability of SNARE complex and prevents the release of secretory vesicles from synapstic plasma membrane [23]. Meanwhile, it does not affect the depolarization-evoked dendritic Ca^2+^ transient [24]. Hence, 100 nM BoTx was added in Cs^+^-based internal saline. We found that BoTx did not affect the induction of DSE (Figure 5A), suggesting that the release of 2-AG is independent of SNARE.

Second question is whether astrocytes are involved in DSE. P2X7Rs are widely expressed in the cerebellum, including Purkinje cells and Bergmann glia cells [25]. Activation of P2X7Rs evokes a rapid and pronounced increase of endocannabinoid production in astrocytes [26]. Is it possible that depolarization-induced ATP release from Purkinje cells triggers the activation of astrocytic P2X7Rs, releases 2-AG and evokes DSE? To address this question, a broad-spectrum antagonist of P2X receptors, PPADS (pyridoxal-phosphate-6-azophenyl-2′-4′-disulfonic acid) (10 µM) was bath-perfused during recordings in WT cells. Our results showed that PPADS did not block DSE (Figure 5B), indicating that P2X7R and astrocytes may be not involved in DSE.

Some evidence suggests that DSE is mediated by a reduction in the presynaptic Ca^2+^ [1], [27]. However, another finding argues that DSE is unrelated to Ca^2+^ entry [28]. Therefore, the CB1R-induced presynaptic signaling underlying DSE is not clear. Presynaptic PKC and PKA are reported to regulate synaptic release and mEPSC frequency [29], [30] and trigger presynaptic long-term potentiation at parallel fiber synapses [31]. We then examined the function of PKC and PKA in parallel fiber DSE. WT cells were continuously treated with PKC-selective inhibitor chelerythrine (10 µM) or PKA-selective inhibitor KT5720 (1 µM) before and during experiments. We found that DSE was successfully induced in the application of both chelerythrine (Figure 5C) and KT5720 (Figure 5D), indicating that presynaptic PKC and PKA are not involved in DSE.

## Discussion

**Figure 6:**
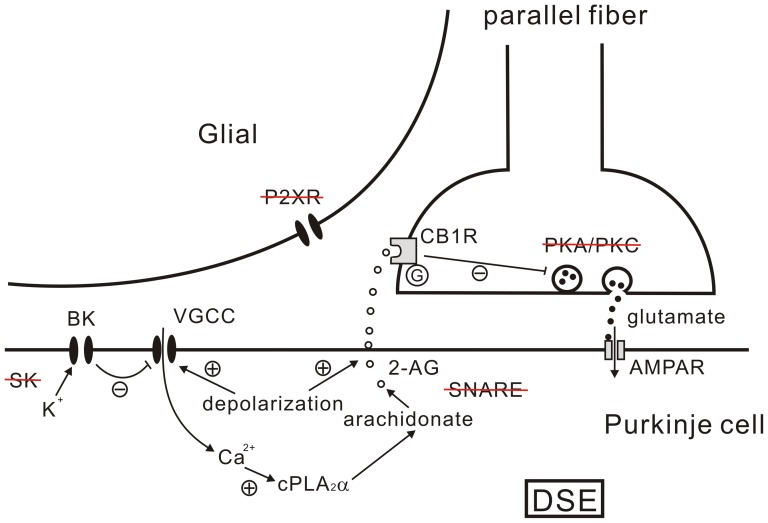
A proposed model for DSE. A proposed model for 2-AG release and DSE at parallel fiber-Purkinje cell synapse. Strikethrough texts show the molecules unrelated to DSE, as demonstrated in the present work. See Discussion for explanation.

The main finding of the present study is that DSE at parallel fiber-Purkinje cell synapse was mediated by the cPLA_2_α/arachidonic acid pathway. DSE was significantly inhibited in cPLA_2_α KO mice and rescued by the application of arachidonic acid in the bath. The action of arachidonic acid in DSE was prevented by MAGL, the degradation enzyme of 2-AG [17], [18], but not FAAH that hydrolyzes AEA [19], [20]. These data first demonstrated that cPLA_2_α/arachidonic acid/2-AG signaling induces DSE at parallel fiber-Purkinje cell synapse, as summarized by a model in Figure 6. As the explanation for this model, postsynaptic depolarization in Purkinje cell triggers Ca^2+^ influx by activating voltage-gated Ca^2+^ channels and causes a transient elevation of [Ca^2+^]_i_. This internal Ca^2+^ elevation is hindered by the presence of intracellular K^+^ and the opening of BK channels [32]. Micromolar levels of [Ca^2+^]_i_ activate cPLA_2_α to liberate arachidonic acid, which produces 2-AG. The latter is released from Purkinje cells into the extracellular space independent of SNARE, diffuses retrogradely and binds to CB1Rs at the parallel fiber terminal. Finally, CB1R triggers a PKA and PKC-independent mechanism to suppress presynaptic glutamate release (DSE).

AEA and 2-AG are derivates of arachidonic acid [33]. They are highly distributed in the hippocampus and cerebellum [34] and considered to be the main endocannabinoids mediating DSE in CNS [35]–[37]. It is generally accepted that the productions of AEA and 2-AG are Ca^2+^-dependent [38], but it is unclear how elevated Ca^2+^ leads to enhanced endocannabinoid production during DSE. PLC/diacylglycerol lipase (DAGL) signaling meditates the formation of 2-AG in hippocampus [38], implying that PLC/DAGL might control the production of 2-AG in DSE. Against to this hypothesis, strong evidence shows that DSE is independent of mGluR1s, PLC and DAGL [36], [39]. The short depolarization in DSE is not strong enough to simulate the PLC/DAGL-dependent 2-AG production [38]. Eicosanoid biosynthesis is highly interactive and often changes among cell signaling pathways on demand [40]. Except the PLC/DAGL signaling, other pathways have been shown to generate the endocannabinoid production [34], [41]. Indeed, glucocorticoid or cyclooxygenase stimulation directly shifts arachidonic acid metabolism toward endocannabinoid synthesis [33], [40]. Although it is impossible to directly detect the biosynthesis of 2-AG from arachidonic acid in DSE that happens within seconds, our MAGL/FAAH experiments clearly showed that 2-AG is the downstream factor of arachidonic acid. Therefore, we conclude that, at parallel fiber-Purkinje cell synapse, the brief depolarization causes the cPLA_2_α activation and shifts the arachidonic acid metabolism towards promoting 2-AG production.

Although DSE has been extensively studied in hippocampal and cerebellar neurons [1]–[4], [15], [37], [42]–[44], several important questions are unsolved. (1) Most retrograde messengers are stored in vesicles and released through exocytosis. How is endocannabinoid released? Does it require any special apparatus? (2) What is the presynaptic mechanism after CB1R is activated? (3) ATP is released from neurons in response to depolarization [45], which may activate astrocytic P2X7Rs and evoke the endocannabinoid production and release [26]. Does P2X7R participate in DSE? Although the present work was mainly focused on the function of cPLA_2_α/arachidonic acid/2-AG signaling in DSE, these questions are also tentatively investigated. Using a series of inhibitors, including BoTX, chelerythrine and KT5720, we showed that DSE is independent of SNARE, PKC and PKA. Although these results were negative, they provide some evidence for future experiments studying precise mechanisms of DSE.

P2X7R is expressed in Purkinje cells and glial cells [25]. Since ATP is released from neurons upon depolarization, we hypothesized that released ATP might activate P2X7Rs on Purkinje cells and glia, and subsequently evoke endocannabinoid production [26]. Unexpectedly, we did not observe inhibition of DSE when we applied PPADS. A previous study reported that ATP release from neurons is crucially dependent on the stimulus frequency [45]. This leads us to propose that the depolarization protocol used in the present work might not be strong enough to stimulate ectopic endocannabinoid release. Alternatively, strong depolarization in Purkinje cells may recruit more ectopic endocannabinoid release and cause more profound inhibition at parallel fiber-Purkinje cell synapse. A current viewpoint suggests that DSE plays a neuroprotective role by suppressing presynaptic glutamate release in response to excitotoxicity and neuronal death [1]–[3], which is strengthened by findings that CB1R KO mice are much more subject than control mice to neurotoxic events [46] and CB1Rs are tonically activated in MAGL knock-out mice [47]. However, our result that DSE usually recovers within 90 s suggests that the neuroprotective role of DSE in neurotoxicity may be overestimated. Future work should be conducted to assess the function of ectopic endocannabinoid release from glia in the process of neurotoxicity.

## Materials and Methods

All experiments were performed according to the guidelines of the National Institutes of Health (USA) regarding the care and use of animals, were approved by the Animal Experimentation Ethics Committee of Zhejiang University, and were specifically designed to minimize the number of animals. Original breeding pairs of the KO strain were obtained from Dr. Adam Sapirstein (The Johns Hopkins University School of Medicine, Baltimore, MD) and maintained at the Experimental Animal Center of Zhejiang University. Mice were kept under temperature-controlled conditions on a 12:12 h light/dark cycle with food and water *ad libitum*.

Electrophysiological experiments were modified from our previous work [48]–[50]. Parasagittal slices of the cerebellar vermis (250 µm) were prepared from P17–23 mice using a vibrating tissue slicer (Leica VT1000S, Germany) and ice-cold standard artificial cerebrospinal fluid (ACSF) containing (in mM): 125 NaCl, 2.5 KCl, 1.25 NaH_2_PO_4_, 1 MgCl_2_, 2 CaCl_2_, 26 NaHCO_3_ and 25 D-glucose, bubbled with 95% O_2_ and 5% CO_2_. After recovery for 30 min at 37°C, slices were placed in a submerged chamber that was perfused at 2 ml/min with ACSF supplemented with 10 µM GABAzine to block GABA_A_ receptors. Recording electrodes were filled with either a Cs^+^-based solution containing (in mM): 135 CsMes, 10 CsCl, 10 HEPES, 4 Na_2_ATP, 0.4 Na_3_GTP, and 0.3 EGTA (pH 7.2), or a K^+^-based solution containing (in mM): 120 Kgluconate, 4 NaCl, 9 KCl, 3.48 MgCl_2_, 10 HEPES, 4 Na_2_ATP, 0.4 Na_3_GTP, 17.5 sucrose (pH 7.2). Resistances of recording pipettes were typically 1.5–3 MΩ, and uncompensated series resistances were <5 MΩ.

Purkinje cells were visualized under an upright microscope (BX51; Olympus Optical, Tokyo, Japan) with a 40× water-immersion objective and equipped with infrared differential interference contrast enhancement. Whole-cell recordings were obtained with an Axopatch 700B amplifier (Molecular Devices, Foster City, CA). Currents were filtered at 1 kHz and digitized at 10 kHz. For parallel fiber stimulation, standard patch pipettes were filled with ACSF and placed in the middle third of the molecular layer. Synaptic responses were evoked every 20 s using 12–16 µA pulses (100 µs duration).

Drugs were purchased from Sigma (St. Louis, MO) and Tocris (Bristol, UK) unless stated otherwise. Data analysis was performed using Excel 2003 (Microsoft, Chicago, IL), Clampfit 10 (Molecular Devices) and Igor Pro 6.0 (Wavemetrics, Lake Oswego, OR). All group data are shown as mean ± SEM. Student's *t* tests were used to determine P values. n represents numbers of cells used in each experiment derived from at least three animals. Cells were excluded from the study if series resistance or input resistance varied by more than 15% over the course of an experiment.

## Supporting Information

Figure S1 **AM251 blocks DSE.** (A) Control (open circles) and test (closed circles) EPSC responses from one WT Purkinje cell plotted over time. Representative EPSCs are shown at right. AM251 was applied in the bath, as indicated by the bar. Stimulus artifacts are blanked for clarity. The percentage inhibition of test EPSCs (87.1±10.7%; n = 16) is shown in (B). *, P<0.05.
(TIF)
