# Correction: Cytosolic Phospholipase A_2_ alpha/Arachidonic Acid Signaling Mediates Depolarization-Induced Suppression of Excitation in the Cerebellum

**DOI:** 10.1371/annotation/d08032c8-4a27-4c9c-8ef7-b3ec64351cde

**Published:** 2012-11-20

**Authors:** De-Juan Wang, Dong Yang, Li-Da Su, Ya-jun Xie, Lin Zhou, Cheng-Long Sun, Yin Wang, Xin-Xin Wang, Liang Zhou, Ying Shen

There was an error in the equal contributions designation. De-Juan Wang, Dong Yang, and Li-Da Su should be listed as co-first authors. 

