# Supplementary figures and images for: Cytosolic Phospholipase A2 alpha/Arachidonic Acid Signaling Mediates Depolarization-Induced Suppression of Excitation in the Cerebellum
Source: PLoS One. 2012 Aug 22;7(8):e41499. doi: 10.1371/journal.pone.0041499 (PMC3425552; doi:10.1371/journal.pone.0041499)

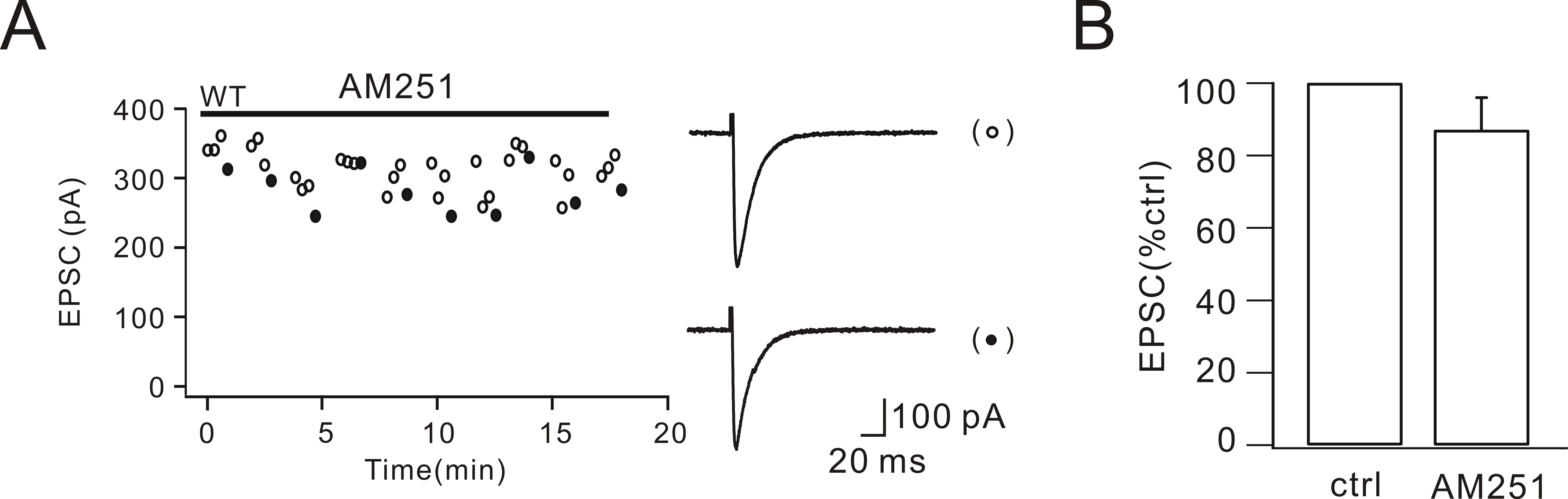

Supplement: Figure S1 — AM251 blocks DSE. (A) Control (open circles) and test (closed circles) EPSC responses from one WT Purkinje cell plotted over time. Representative EPSCs are shown at right. AM251 was applied in the bath, as indicated by the bar. Stimulus artifacts are blanked for clarity. The percentage inhibition of test EPSCs (87.1±10.7%; n = 16) is shown in (B). *, P<0.05. (TIF) [file pone.0041499.s001.tif]
